# Comprehensive Quality Assessment of Refractory Materials Used in Aluminum Production

**DOI:** 10.3390/ma18173957

**Published:** 2025-08-24

**Authors:** Miriam Andrejiova, Štefan Markulik, Miriama Pinosova, Marek Šolc

**Affiliations:** 1 Department of Applied Mathematics and Informatics, Faculty of Mechanical Engineering, Technical University of Košice, 042 00 Košice, Slovakia; miriam.andrejiova@tuke.sk; 2 Department of Quality, Safety, and Environment, Faculty of Mechanical Engineering, Technical University of Košice, 042 00 Košice, Slovakia; miriama.pinosova@tuke.sk; 3 Institute of Metallurgical Technologies and Digital Transformation, Faculty of Materials, Metallurgy and Recycling, Technical University of Košice, 042 00 Košice, Slovakia; marek.solc@tuke.sk

**Keywords:** refractory materials, infiltration, NaCl–KCl melts, corrosion resistance

## Abstract

Corrosion of refractory materials in NaCl–KCl melts is a major issue affecting the service life of linings in aluminum metallurgy, where these salts serve as the basis for covering and refining mixtures. The aim of this study was to comprehensively evaluate the corrosion resistance of alumina-silicate refractory materials (ASRM) with a high SiO_2_/Al_2_O_3_ ratio in contact with melts of varying NaCl–KCl ratios. Static crucible corrosion tests were conducted in accordance with the technical specification CEN/TS 15418:2006. Macro- and microscopic analysis, chemical analysis (AAS), and semi-quantitative EDX analysis enabled detailed monitoring of the depth of melt infiltration, microstructural changes, and element distribution within the material. The results demonstrated that as the NaCl content in the melt increased, there was a significant rise in both the depth of infiltration and the degree of material degradation. A linear regression model confirmed a very strong positive correlation between NaCl content and the extent of damage (*R*^2^ = 0.967). Chemical analysis revealed that the silicon content decreases in the infiltrated zone, while aluminum remains stable, indicating superior corrosion resistance of Al_2_O_3_ compared to SiO_2_. EDX analysis also confirmed increased concentrations of sodium and chlorine in the infiltrated areas, complementing the AAS results and providing more precise mapping of the distribution of corrosion products within the material structure. These findings provide a quantitative basis for optimizing the composition of refractory materials and designing protective strategies to extend their service life under the aggressive operating conditions of aluminum production.

## 1. Introduction

The degradation of refractory materials in NaCl–KCl melt environments is a significant problem in the metallurgical industry, particularly in aluminum production. The failure of these materials results in frequent downtime, increased maintenance costs, and potential environmental risks. Therefore, effective protection and optimization of refractory linings are crucial for the safe and economical operation of production technologies.

Refractory materials are specialized construction materials designed for use at high temperatures and in aggressive chemical environments, such as those encountered in aluminum smelting. NaCl–KCl melts are typical covering and refining salts used in metallurgy, which can cause chemical corrosion of furnace linings. In this context, corrosion refers to the chemical or physicochemical degradation of a material due to contact with a melt. Consequently, investigating the degradation of refractory materials in NaCl–KCl melt environments is a key topic in materials engineering.

The primary function of these materials is to ensure the structural integrity and chemical resistance of furnace linings, electrolytic baths, and refining units. Corrosion caused by chloride-based melts is one of the most serious degradation mechanisms affecting the service life and reliability of these materials.

In recent decades, the issue of corrosion of refractory materials in contact with molten metals has become the subject of intensive scientific research. Pioneering work by Farris & Allen [1] demonstrated that sodium present in molten metal in the form of sodium oxide (Na_2_O) reacts rapidly with alumina-silicate refractory materials, causing significant degradation of their structure. Research [2] confirmed that the interaction of molten salts with different Na_2_O contents and mullite leads, depending on the chemical composition of the melt, to the formation of either Al_2_O_3_ or compounds in the Na_2_O–Al_2_O_3_–SiO_2_ system. Kennedy [3] in his experiments exposed mullite to the action of soda at a temperature of 1000 °C for 125 h and observed the formation of carnegieite (NaAlSiO_4_) and sodium β-alumina (NaAl_11_O_17_) phases. He identified volume expansion resulting from the formation of these phases as the main mechanism of degradation.

The chemical composition, especially the SiO_2_/Al_2_O_3_ ratio, plays a crucial role in the corrosion resistance of alumina-silicate refractory materials in contact with Al-Mg melts. Barandehfard et al. [4] demonstrated that increasing the SiO_2_ content leads to greater corrosion depth, while a higher Al_2_O_3_ content improves the resistance of the material. However, this effect is not linear—at certain concentrations, further increases in SiO_2_ have no significant effect on the latent phase of corrosion. The interaction of chloride-based melts with linings has been investigated using laboratory static crucible tests and under industrial conditions. Standard laboratory tests using the static crucible method, as described in this work, have been used for decades to quantify the dissolution of Al-Si phases at temperatures of 650–900 °C [5,6,7].

Several studies indicate that the main cause of degradation of refractory linings is the infiltration of low-viscosity chloride melts into the porous structure of these materials [6,8,9]. In recent years, the use of nanoparticles and ceramic composites has brought about a significant shift in the development of refractory linings [10,11,12,13]. Recent studies have focused on comparing different types of refractory materials with varying chemical compositions and on elucidating the mechanisms of their corrosion. Wang et al. [14] investigated the corrosion behavior of high-purity α-Al_2_O_3_ refractory materials in contact with soda-based glass melts under conditions simulating actual operation. The results, confirmed by XRD and SEM-EDS analyses, showed that the phase composition and microstructure at the interface have a decisive influence on the corrosion resistance of these materials. The influence of operating parameters such as temperature, exposure and chemical composition of the melt was systematically investigated in experiments and model studies. Tran et al. [15] demonstrated that higher temperatures significantly increase the depth of melt infiltration into refractory materials, while the effect of exposure time is less significant. Using regression analysis, they quantified the influence of parameters such as Fe_2_O_3_ content and temperature on penetration depth, enabling the optimization of both material composition and operating conditions. The corrosion behavior of metallic materials in chloride-based melt environments is also the subject of intensive research, as these materials are often used in the manufacture of load bearing and sealing components [16,17,18].

Collectively, these findings provide a comprehensive basis for understanding the interactions between Al-Si refractory materials and chloride melts. The combination of chemical thermodynamics, microstructural characteristics, and pore system optimization plays a key role in these processes. Most previous research has focused on other types of melts or refractory materials with different chemical compositions. Knowledge of the interaction between high-purity SiO_2_/Al_2_O_3_ refractory materials and NaCl–KCl melts remains limited, highlighting the need for further investigation in this area. In contrast to studies focusing on MgO-based materials, Ni alloys, silicon carbide (SiC), and others, this work specifically addresses high-purity SiO_2_/Al_2_O_3_ systems.

Addressing the issue of refractory material degradation requires a multidisciplinary approach, combining materials engineering, physical chemistry, thermodynamics, and the optimization of operating parameters.

This experimental research was initiated at the request of an industrial partner that lacks the technical equipment required to perform experimental tests to verify the corrosion resistance of refractory materials in contact with NaCl–KCl melts. The study aims to enhance understanding of the corrosion resistance of alumina-silicate refractory materials in contact with NaCl–KCl melts through a comprehensive evaluation of their behavior. Using analytical methods, statistical modeling, and comparison with existing literature data, we have attempted to identify the main factors influencing the degradation of these refractory materials. Our findings are intended to contribute to extending the service life of alumina-silicate refractory materials under the aggressive operating conditions of aluminum production.

## 2. Materials and Methods

The experimental part of this study focuses on evaluating the corrosion resistance of alumina-silicate refractory materials in contact with chloride-based melts commonly used in the production and refining of pure aluminum. The samples examined belong to the category of alumina-silicate refractory materials (hereinafter referred to as ASRM) with a high content of silicon dioxide (SiO_2_) and aluminum oxide (Al_2_O_3_). These materials are ideal for applications requiring high resistance to chemical corrosion and moderate thermal load. Their composition and internal structure are similar to those of materials used in aluminum processing equipment, where they frequently come into contact with very hot and chemically aggressive molten metals. A two-component high-temperature salt mixture of sodium chloride (NaCl) and potassium chloride (KCl) serves as a typical corrosive medium (melt) in metallurgical processes for aluminum production, where materials are exposed to aggressive melts during operation. Corrosion tests were performed using a static crucible method, which allows evaluation of the interaction between refractory materials and aggressive chloride-based media under standardized laboratory conditions. As part of the experimental procedure, exposure parameters (temperature, time, concentration of corrosive media) were precisely controlled to simulate operating conditions. After corrosion testing, the samples underwent comprehensive macro- and microstructural analysis (ZEISS Neophot 32 microscope—Carl Zeiss Jena GmbH, Jena, Germany), chemical analysis (Perkin Elmer 3100—PerkinElmer, Inc., Waltham, MA, USA), and semi-quantitative EDX (energy-dispersive X-ray) analysis (JEOL 35CF microscope—JEOL Ltd., Akishima, Japan) to identify, characterize, and quantify structural changes in the materials.

### 2.1. Characterization and Selection of Input Materials and Corrosive Media

The characteristics of raw materials are a key element in any experimental work focused on evaluating material properties. Proper selection, thorough analysis, and appropriate treatment of raw materials have a significant impact on the final quality, reliability, and corrosion resistance of refractory materials. For refractory materials, it is important to consider not only their chemical and phase composition, but also the technological parameters of their production, which determine their microstructure and final properties. The choice of a suitable corrosive medium is equally important, as it must accurately represent real operating conditions and allow for an objective evaluation of the interactions with the tested material. The detailed characterization of both the refractory materials and corrosive media forms the basis for the design and implementation of subsequent experimental tests.

As part of the experimental evaluation, shaped refractory materials based on aluminum oxide (Al_2_O_3_) and silicon oxide (SiO_2_) were designated as ASRM. Phase and chemical analyses were performed to assess these materials in detail, and the laboratory results were compared with the manufacturer’s data. The phase composition was determined using the MIKROMETA 2 X-ray diffractometer (XRD) (30 kV, 12 mA, 1000 imp./s, Cu Kα radiation; CHIRANA Praha, a.s., Prague, Czech Republic) with a scanning speed of 2°/min in the range of 5–110°. This method enabled the identification of the main crystalline phases, especially mullite, which is characteristic of alumina-silicate refractory materials. Chemical composition was analyzed by AAS. The determined contents of major and minor elements were compared with the manufacturer’s data to assess any deviations that could influence the corrosion resistance of the material in a NaCl–KCl melt environment. Key physical and mechanical properties, such as cold compressive strength, bulk density, apparent porosity, water absorption, and maximum operating temperature, were determined based on both manufacturer data and laboratory measurements. These parameters are critical for evaluating the material’s performance under metallurgical conditions.

A mixture of sodium chloride (NaCl) and potassium chloride (KCl) from Lach-Ner s.r.o. (Neratovice, Czech Republic), supplied by ITES Vranov, s.r.o. (Vranov nad Topľou, Slovakia), was used as the corrosive medium. The experimental conditions were designed to simulate real operating temperature profiles (770–801 °C) and exposure times (24–96 h) typical of industrial processes.

### 2.2. Combined Analytical Methodology for Evaluating Corrosion Processes

As part of the experimental investigation, we first performed macroscopic analysis, followed by microscopic imaging to obtain a more detailed view. These observations were subsequently complemented by chemical analysis and semi-quantitative EDX analysis.

**Figure 1:**
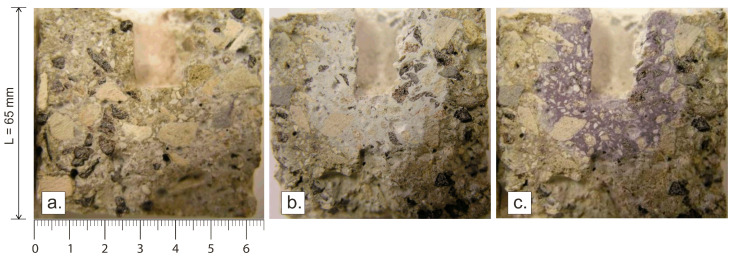
Macroscopic identification of chloride penetration zones after application of a diluted silver nitrate (AgNO_3_) solution. (**a**) Control condition—the sample surface without AgNO_3_ application exhibits minimal contrast between corroded and pristine areas.; (**b**) after AgNO_3_ application—distinctive delineation of penetration zones with clear boundaries is visible; (**c**) after AgNO_3_ application and UV exposure, maximum contrast is achieved, allowing precise measurement of chloride infiltration depth.

1. Macroscopic analysis (Figure 1) focused on observing changes on the sample surfaces and identifying visible signs of corrosion.
  − Identification of Chloride Penetration: To enable reliable macroscopic evaluation of chloride penetration after corrosion tests, it was necessary to optimize the visualization method. Initially, 1 wt.% (0.1 g) of colored chloride salts (CoCl_2_—blue, CuCl_2_—pink, FeCl_3_—brown, NiCl_2_—yellow) were added to 10 g of NaCl, which imparted a characteristic color to the melt. However, after corrosion testing, the resulting color intensity was insufficient to clearly distinguish areas of chloride penetration. At concentrations of colored chlorides above 50 wt.%, significant alterations in the composition of the corrosive medium occurred, rendering this method unsuitable. As an effective alternative, the application of a 1% silver nitrate (AgNO_3_) solution was employed, providing a clear and detectable reaction with chloride ions.
  − Measurement of Cl^−^ Ion Depth Infiltration Using AgNO_3_: A 1% silver nitrate (AgNO_3_) solution was applied to the sample cross-sections, resulting in the formation of a white silver chloride (AgCl) precipitate on the surface (Figure 1b). This visually indicated the depth of chloride penetration into the material. Under UV radiation, the precipitate darkened (Figure 1c), further highlighting regions infiltrated by chlorides. The cross-section for this evaluation was prepared after the corrosion test.
2. Chemical analysis was performed using AAS. Samples were collected from specific locations within the corrosion-damaged masonry using a 2 mm diameter drill bit at a controlled sampling depth (±0.1 mm). Sampling points were defined at three levels relative to the bottom of the crucible (reference point, x = 0): 10 mm (Location–*1*), 25–35 mm (Location–*2*; depending on the sample being assessed) and 45 mm (Location–*3*).
3. Semi-quantitative elemental analysis were conducted on an area of approximately 100 μm × 100 μm.

This approach facilitated a comprehensive evaluation of the interactions between the material and the melt (or corrosive medium), while systematic reproducibility and data validation were essential for the effective optimization of refractory systems in industrial applications.

### 2.3. Static Crucible Method for Evaluating Corrosion in NaCl–KCl Melt

Static crucible corrosion tests were performed to evaluate the resistance of ASRM refractory materials from the Al_2_O_3_–SiO_2_ system to NaCl–KCl chloride melts. The crucibles used in the tests were prepared from shaped refractory samples based on the Al_2_O_3_–SiO_2_ system. These refractory blocks were cut using a diamond saw into specimens measuring 65 mm × 65 mm × 76 mm. In each block, a hole with a diameter and depth of 25 mm was drilled and subsequently filled with the dried chloride mixture (12–15 g) serving as the corrosive medium. The test temperature of 801 °C was selected based on the melting temperature of the medium and industrial requirements for aluminum metallurgy, ensuring a homogeneous melt throughout the exposure period. The corrosion tests were conducted in a laboratory box electric resistance furnace (SNOL 8.2/1100, UAB SNOL, Utena, Lithuania) at atmospheric oxygen pressure (pO_2_ ≈ 212.78 kPa). The furnace temperature was accurately maintained at 801 °C throughout the experiment. Samples were heated at this temperature for 96 h, simulating typical industrial operating conditions. After free cooling, the samples were sectioned with a diamond saw for cross-sectional analysis.

The samples were analyzed in three main steps:

1. Macroscopic analysis involved visual assessment of chloride infiltration depth on the cross-sections, supplemented by optical microscopy in reflected light for detailed imaging of selected areas.
2. Chemical analysis was performed to quantify chloride and other relevant element contents.
3. Electron microscopy was conducted using a JEOL 35CF microscope, with chloride infiltration monitored by semi-quantitative EDX analysis.

This integrated approach enabled a comprehensive evaluation of the degradation mechanisms of the refractory materials under investigation, including quantification of the influence of chlorides on their integrity and stability at high temperatures. The results obtained provide a basis for optimizing the composition of materials intended for use in aggressive melt environments.

### 2.4. Statistical Methods for Evaluating NaCl–KCl Melt Infiltration

The evaluation and analysis of the obtained data were performed using basic statistical methods, specifically descriptive statistics and statistical hypothesis testing. Regression and correlation analysis were used to identify the type and degree of dependence between the analyzed variables. When testing statistical hypotheses, decisions to accept or reject the null hypothesis were based on the *p*-value: if the *p*-value was less than the chosen significance level (*α*), the null hypothesis was rejected in favor of the alternative hypothesis; if the *p*-value was equal to or greater than *α*, the null hypothesis was not rejected.

Regression analysis is among the most widely used statistical methods; its primary objective is to determine a mathematical function—termed a regression function or regression model—that best describes the relationship between observed quantitative variables.

The analysis within our research focused on a simple regression model (see Equation (1)), where the relationship between one independent (input) variable *X* and one dependent (output) variable *Y* is examined. This model can generally be expressed as follows:

(1) $$Y=f\left(X\right)+\varepsilon .$$

The regression function *f* represents the relationship between the independent variable *X* and the dependent variable, where *ε* denotes the random error of the model, which represents the effect of random influences and other factors not included in the model. The regression function can take various mathematical forms. In this study, we consider both linear and nonlinear regression models, including polynomial, hyperbolic, exponential, and logarithmic models. The quality of the model will be verified using the F-test of statistical significance of the model, in which we test at a significance level of *α* = 0.05 the null hypothesis H_0_: the regression model is not statistically significant against the alternative hypothesis H_1_: the regression model is statistically significant.

The strength of the relationship between the analyzed variables was evaluated using Pearson correlation coefficient *r* for a linear regression model and using the correlation index *I* for a nonlinear regression model. Pearson correlation coefficient *r* takes values in the range ⟨−1; 1⟩. A positive value of the coefficient indicates a direct linear dependence between the variables, while a negative value indicates an indirect linear dependence. The following scale of absolute values of the correlation coefficient was used to evaluate the strength of the linear relationship: no correlation (|*r*| < 0.29), weak correlation (0.30 < |*r*| < 0.49), moderate correlation (0.50 < |*r*| < 0.79), strong correlation (0.80 < |*r*| < 1). The correlation index *I*, used in nonlinear models, takes values in the range ⟨0; 1⟩. The closer its value is to 1, the closer the dependence between the variables is and the better it is captured by the selected model.

The coefficient of determination *R*^2^ (for a linear model) or the index of determination *I*^2^ (for a nonlinear model), which take values in the range ⟨0; 1⟩, quantify the proportion of the variability of the dependent variable that can be explained by the independent variables using the regression model under consideration. The closer the value of *R*^2^ (or *I*^2^) is to 1, the closer the relationship between the dependent and independent variables is, and the model explains a larger part of the variability of the dependent variable.

The data obtained were statistically processed using software tools, namely Minitab version 18 (regression analysis), R version 4.4.3 (Stat package), and OriginPro 2019b for graphical processing of the results.

## 3. Results and Discussion

As discussed in the Introduction, corrosion in chloride melt environments critically influences the durability of refractory materials. As part of the experiment, five samples of refractory material (ASRM-*1* to ASRM-*5*) were exposed to melts with different molar ratios of NaCl–KCl, ranging from pure NaCl (100%) and KCl (100%) to mixtures with various ratios (20/80%, 50/50%, and 80/20%).

The methodology was based on the static crucible method according to technical specification CEN/TS 15418:2006 [19], monitoring macroscopic changes, microstructure and chemical composition in corroded areas using AAS and EDX. The results provide a detailed insight into the relationship between the NaCl/KCl ratio and the formation of reaction layers, which allows the composition of refractory materials for aggressive environments to be optimized. The experiment identified fundamental changes in the chemical composition between the melt and the refractory material, which have a significant impact on the long-term stability of refractory linings (lining bricks) used in industrial furnaces.

### 3.1. Composition and Corrosion Behavior of Refractory Materials: Analytical Results

The results of phase and chemical analysis of refractory materials designated as ASRM are shown in Table 1. The values presented in the table were obtained directly from the manufacturer and subsequently verified by laboratory measurements. The use of XRD and AAS methods ensured high reliability of the results, which allows for the correction of production specifications.

**Figure 2:**
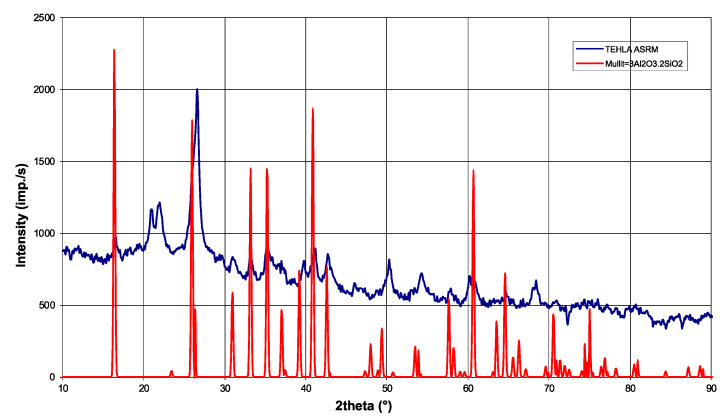
X-ray diffractogram of the refractory material, showing identified mullite phases.

Phase analysis confirmed that the dominant components are silicon dioxide (SiO_2_) and aluminum oxide (Al_2_O_3_), while the presence of mullite was also identified in the analyzed samples as the main crystalline phase (Figure 2), which is typical for alumina-silicate refractory materials. The measured value of SiO_2_ (65%) is 3% lower than the manufacturer’s specification (68%). Al_2_O_3_ shows complete agreement (26%), confirming the consistent presence of aluminum in the material. Fe_2_O_3_, with a measured value of 2%, meets the manufacturer’s specification (<2.5%), which complies with the material purity requirements. Laboratory measurements also supplemented missing data on minor oxides such as Fe_2_O_3_, TiO_2_, CaO, MgO, Na_2_O, and K_2_O. The difference in SiO_2_ is typical for ceramic materials. Optimizing homogenization and firing control can minimize variability, but complete elimination is prevented by the natural diversity of the raw materials.

The chemical analysis confirmed that the sample mainly contains silicon (Si) and aluminum (Al), with the laboratory-determined content of these elements being 29.43% for Si and 13.76% for Al. The iron (Fe) content in the sample is 1.59%, which is lower than the maximum value of 1.75% declared by the manufacturer. Other elements, such as titanium, calcium, magnesium, sodium, and potassium, are not listed by the manufacturer in its data, but laboratory analysis confirmed their presence in trace amounts ranging from 0.22% to 1.08%. The presence of key components—silicon dioxide (SiO_2_) and aluminum oxide (Al_2_O_3_)—confirms that the material meets the basic requirements for alumina-silicate refractory materials and is suitable for applications requiring high corrosion resistance and stability at extreme temperatures.

Selected physical and mechanical properties of the refractory material, provided by the manufacturer and verified by laboratory measurements, are summarized in Table 2. The parameters monitored included cold compressive strength, bulk density, apparent porosity (i.e., the proportion of pores in the material), water absorption, and maximum operating temperature.

The values presented in Table 2 represent average measurements from 5 samples, supplemented by calculated standard deviations that illustrate the variability of properties among the individual samples. This variability is a natural consequence of manufacturing differences, local material heterogeneity, and minor deviations in the measurement method. The average compressive strength reaches 45 MPa with a standard deviation of 2 MPa. This value is lower than the declared strength by the manufacturer, which is 60 MPa. The low standard deviation indicates good repeatability of the measurements. The discrepancy between the measured and declared strength may be caused by the presence of microdefects or material inhomogeneities. Lower strength is often associated with increased porosity, which can lead to greater permeability to aggressive substances and potentially deteriorate corrosion resistance. However, the determination of these effects was not the subject of our current measurements and represents an important topic for further research. The bulk density is 2130 ± 7.7 kg/m^3^, close to the declared value of 2150 kg/m^3^. The apparent porosity is 17 ± 1.3%, slightly higher than the declared 14%, which may affect strength and water absorption. The water absorption is 8.1 ± 0.58%, with no declared value provided. The maximum operating temperature was not measured; the declared value is 1100 °C.

These properties were verified by laboratory measurements in accordance with the relevant standards and standard methods. Cold compressive strength was determined using a Zwick/Roell Z050 universal compression testing machine (Zwick GmbH & Co. KG, Ulm, Germany). The bulk density was calculated based on the ratio of weight to volume of the sample, while the apparent porosity was determined by the liquid immersion method. The water absorption of the material was determined as the percentage increase in weight after absorption of water. The maximum operating temperature was taken from the manufacturer’s data, which is based on long-term thermal tests and industrial applications of the material.

In addition to the physical parameters mentioned, an important characteristic of refractory materials is their thermal conductivity (bulk thermal conductivity). This property significantly influences the temperature distribution within the material during operation, which directly impacts local corrosion processes and mechanical stresses at the interfaces between different materials or individual phases of the lining. Temperature discontinuities at the interface, caused by differences in thermal conductivity values, acoustic mismatch, and chemical properties, can lead to the formation of microcracks and increased infiltration of molten salts, thereby significantly accelerating corrosion processes [20].

Typical values of thermal conductivity for aluminosilicate refractory materials (ASRM) range approximately from 1 to 2 W·m^−1^·K^−1^ at room temperature, and these values may increase further with rising temperature. Including these data in the comprehensive characterization of the material allows for a better understanding of its behavior at high temperatures and enhances corrosion resistance in aggressive NaCl–KCl melt environments. Inclusion of thermal conductivity values and analysis of the nature of interfaces in relation to operational conditions represents a key step in optimizing refractory materials for applications in the aluminum industry.

These results provide a basis for further evaluation of corrosion resistance and interpretation of corrosion test results in the context of requirements for refractory materials in the metallurgical industry.

**Figure 3:**
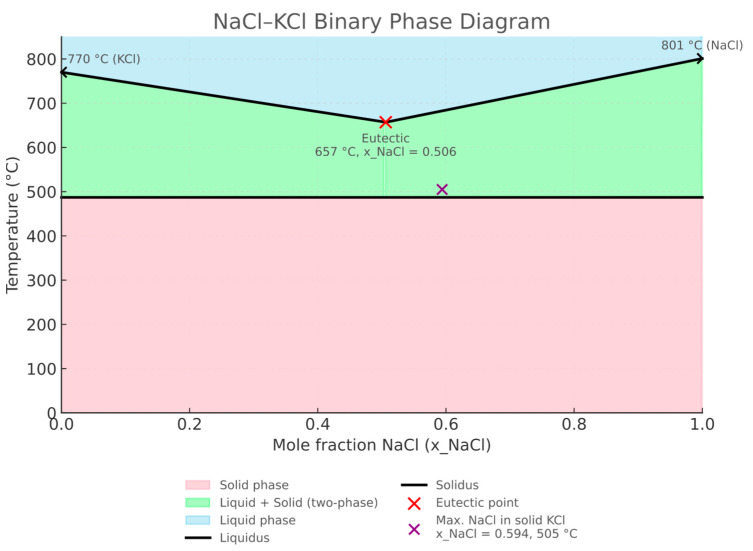
Binary phase diagram of the NaCl–KCl system. The liquidus (black) and solidus (black) boundaries delimit three regions: L—fully liquid (blue), L + S—liquid + solid coexistence (green), and S—fully solid (pink). The eutectic occurs at NaCl = 0.506 and 657 °C (red symbol). Melting points of the pure components are 770 °C (KCl) and 801 °C (NaCl). The maximum solubility of NaCl in solid KCl is NaCl = 0.594 at 505 °C (purple symbol).

The phase diagram of the NaCl–KCl system (Figure 3) shows unlimited liquid miscibility above 665 °C and limited solid-state miscibility below 487 °C. The eutectic point is located at 0.506 mole fraction of NaCl, with a melting point of 657 °C, while the maximum solubility of NaCl in the solid solution is 0.594 mole fraction at 505 °C. In the experiments, melts with various NaCl–KCl mass ratios were used, allowing detailed investigation of how melt composition influences the corrosive aggressivity toward refractory materials.

The key physical properties of the chlorides used, which are relevant for assessing their behavior under corrosive conditions, are summarized in Table 3. These data provide a reliable basis for evaluating the effect of NaCl–KCl melt composition on the corrosion resistance of refractory materials in environments simulating actual metallurgical processes.

### 3.2. Behavior of NaCl–KCl Melt with Different Compositions

The samples were exposed to NaCl–KCl melts with different molar ratios (see Table 4), with the temperature and exposure time set and maintained at a constant value throughout the experiment. Based on the weights of the samples before and after the corrosion test, the percentage change in the weight of the NaCl–KCl melt was determined. The parameters analyzed included (1) the mass and molar fraction of chlorides in the melt (see Table 4), (2) the percentage weight loss of the melt after the test.

Table 4 summarizes the results of corrosion tests on ASRM samples in NaCl–KCl melts at a temperature of 801 °C for 96 h. The melt loss (wt.%) increased with increasing NaCl content in the mixture. The lowest melt loss was recorded for the sample with pure KCl (ASRM-*1*), where it reached 33.34%. On the contrary, the highest loss (40.00%) was observed for the sample with pure NaCl (ASRM-*5*). Samples with mixed proportions of KCl and NaCl, specifically ASRM-*2* to ASRM-*4*, showed melt loss ranging from 34.18% to 38.72%, with the loss increasing with increasing NaCl content. Sample ASRM-*3*, which is an equimolar mixture of NaCl and KCl (56.00% KCl and 44.00% NaCl), showed a melt loss of 36.15%. This value is in the middle of the observed range, indicating that even with nearly equal proportions of both salts, the corrosion wear remains substantially higher than that observed for mixtures with a predominance of KCl. This result is important for industrial practice because it points to the need for increased attention when using melts with a similar composition, as the corrosive aggressiveness of the melt is already significant in this case.

The results obtained are consistent with the conclusions of several studies [21,22,23] that evaluated the corrosion resistance of refractory and metallic materials in NaCl–KCl melts. As part of study [24], Ni–Mo–Cr alloy and 316 steel were exposed to a NaCl–KCl melt with added AlCl_3_ at 400 °C for 168 h. While the surface of the Ni–Mo–Cr alloy showed only minimal corrosion damage (small pits), selective corrosion was observed on the 316 steels: iron and chromium were leached out, forming a distinct nickel layer (6.5 μm) exposing the grain boundaries of the material. Vuelvas-Rayo et al. [25] demonstrated that the corrosion activity of high-chromium cast irons (18–30% Cr, 3.8–5.2% C) in NaCl–KCl at 670 °C is primarily determined by the Cr/C ratio. The study [8] demonstrated an exceptional resistance of MgO to NaCl, Na_2_CO_3_ and Na_2_SO_3_ at 600–1200 °C without formation of corrosion products. They experimentally (thermodynamic calculations, XRD) confirmed that MgO is not chemically attacked, unlike Al_2_O_3_ or Cr_2_O_3_, which degrade significantly.

The results of the experiments clearly confirm that the corrosive aggressiveness of the NaCl–KCl melt increases significantly with increasing NaCl content in the mixture. Industry should therefore pay increased attention not only to the selection of suitable materials, but also to the optimization of the melt composition in order to minimize corrosion damage to equipment, with the selection of materials or protective measures being a priority in such conditions.

### 3.3. Macroscopic Analysis and Depth of Melt Infiltration in Refractory Samples

Macroscopic analysis is a key methodology for evaluating the behavior of refractory materials in contact with melts, enabling the identification of processes affecting their service life through the observation of changes visible to the naked eye or under low magnification. The depth of melt infiltration is a critical parameter for evaluating the corrosion resistance of materials, depending on the radius of open pores, surface tension, contact angle, melt viscosity, and contact time. The research confirms that the temperature has a significant effect on the depth of infiltration, while the exposure time did not show a statistically significant effect [15,26,27].

#### 3.3.1. Depth of Melt Infiltration Along the Outer Walls of the Samples

**Figure 4:**
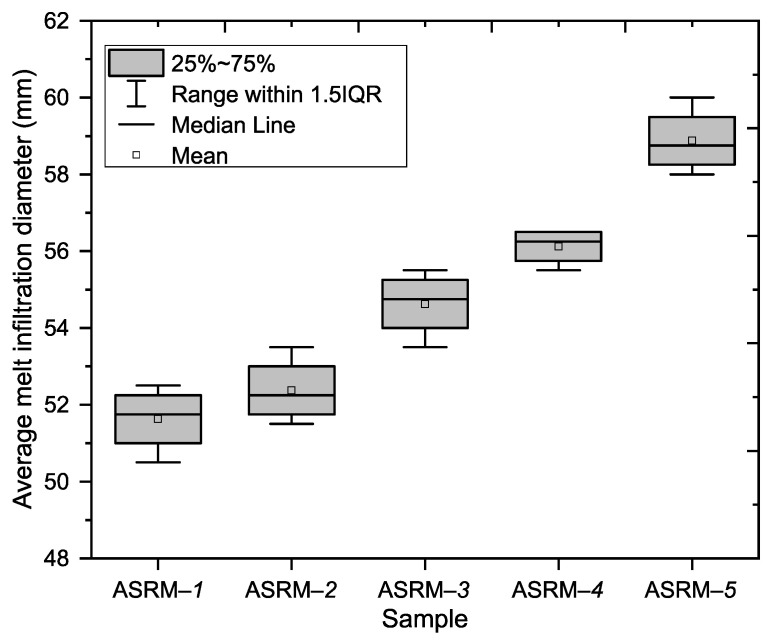
Boxplot—deep penetration of melt on outer walls.

This part of the experiment focused on evaluating the depths of infiltration along the outer walls of the tested samples, with the aim of identifying differences in the degree of penetration depending on the composition of the melt. The melt infiltration diameters were determined for five samples. The melt infiltration depth measurements were performed on four walls of each sample, with the results showing a clear trend of gradual increase in the average infiltration depth from sample ASRM-*1* to sample ASRM-*5* (see Figure 4).

**Figure 5:**
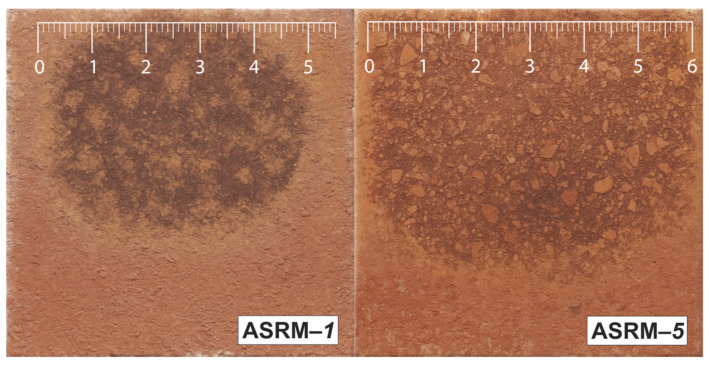
View of the outer walls of samples ASRM–*1* and ASRM–*5* after corrosion testing.

The lowest average infiltration depth on the outer walls (Figure 5) was recorded in sample ASRM-*1* (100% KCl: 51.63; SD [50.266; 52.984]), while the highest value (Figure 5) was found in sample ASRM-*5* (100% NaCl: 58.88; SD [57.516; 60.234]). This increase represents approximately 14.1% compared to sample ASRM-*1*. An increasing trend was observed between the individual samples. The average values of the other samples were as follows: ASRM-*2* (52.38; SD [51.016; 53.734]), ASRM-*3* (54.63; SD [53.266; 55.984]) and ASRM-–*4* (56.13; SD [55.363; 56.887]).

The results of the experiment show that with increasing NaCl content in the melt, the depth of infiltration into ASRM gradually increases. This trend is caused by the higher corrosive aggressiveness of NaCl compared to KCl, which is mainly related to the lower eutectic temperature of the NaCl–KCl system, the greater mobility of Na^+^ ions in the melt, and the formation of crystallization pressures during salt segregation in the pore system of the SiO_2_/Al_2_O_3_. The observed dependence between the composition of the melt and the degree of material damage provides an important basis for evaluating the suitability of refractory materials in environments with high corrosion loads. In areas with a predominance of NaCl, such as aluminum electrolysis, it is advisable to consider the use of materials with higher resistance (e.g., tantalum [28], ceramic composites [29], nanoparticles [11], and similar materials), or the implementation of additional protective measures such as anti-corrosion coatings, varnishes, waxes, ceramic or metal coatings (e.g., based on Zn, Al, Cr), as well as regular maintenance and monitoring of the condition of these protective layers.

A simple regression model was used to evaluate the relationship between NaCl concentration and melt infiltration depth along the outer walls of the sample. In this model, the amount of NaCl is the independent variable (*X*) and the depth of infiltration is the dependent variable (*Y*). It was found that the most appropriate description of this relationship is a linear model in the following form:

(2) $$Y=\beta_{0}+\beta_{1}X+\varepsilon ,$$

where *β_0_*, *β*_1_ represent model parameters (coefficients) and ε denotes random model error. The model (see Equation (2)) allows capturing the dependencies between the amount of NaCl and the melt infiltration rate, which is important for accurate predictions and interpretation of experimental results in the context of evaluating the corrosion resistance of refractory materials.

**Figure 6:**
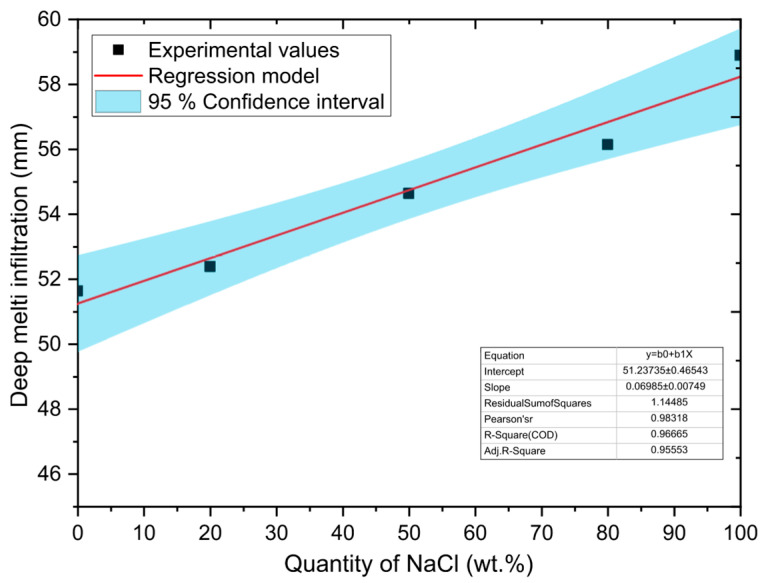
Dependence of melt depth infiltration on NaCl content.

The least squares method was used to estimate the model parameters. Point estimations of the parameters together with 95% confidence intervals are shown in Table 5. A graphical representation of the regression model, including 95% confidence intervals, is shown in Figure 6.

The F-test was applied to verify the statistical significance of the linear regression model. The tested null hypothesis *H*_0_ assumed that the model is not statistically significant, while the alternative hypothesis *H*_1_ represented the opposite statement. The test was performed at a significant level of *α* = 0.05. The results showed that the model has high statistical significance with F = 86.946, *p*-value = 0.0026 < α, which allows *H*_0_ to be rejected in favor of *H*_1_. This finding confirms that the linear model significantly contributes to explaining the variability of the data.

**Figure 7:**
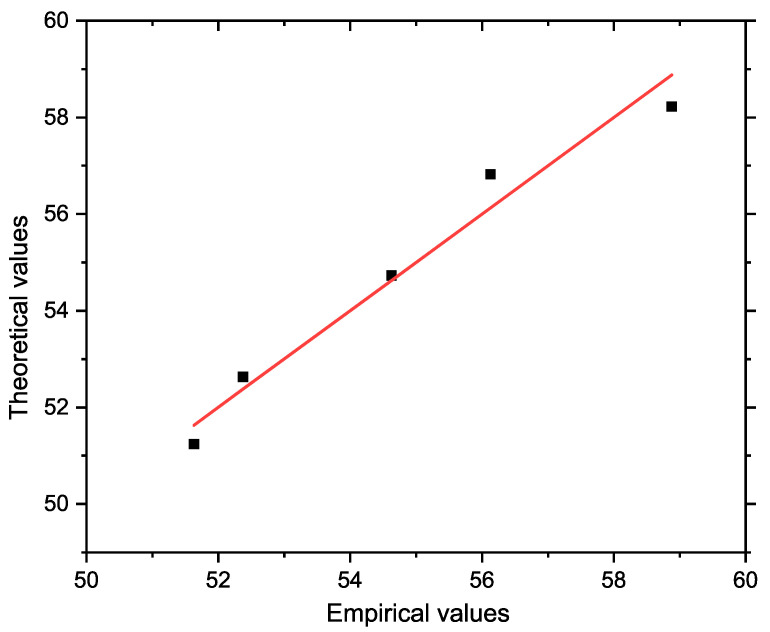
Comparison of actual and predicted values of melt infiltration depth based on a regression model.

The Pearson correlation coefficient (*r* = 0.983) indicates a very strong direct linear relationship. The strength of the relationship between the variables studied was assessed using the coefficient of determination *R*^2^, which reached a value of 0.967. This means that the proposed linear model explains up to 96.7% of the total variability of the dependent variable, i.e., the depth of melted infiltration along the outer walls of the sample. Only 3.3% of the variability remains unexplained by this model, which indicates an extremely strong linear dependence between the amount of NaCl and the depth of infiltration. With increasing NaCl content, there is a clear increase in the depth of chloride melt penetration into the refractory material. At the same time, these results confirm that the model created (see Equation (2)) is also suitable for predicting the values of depth of infiltration depending on the composition of the NaCl–KCl melt. Figure 7 shows a graph comparing the actual and model-predicted (theoretical) values of the depth of infiltration. The *x* axis shows the actual values, and the *y* axis shows the predicted values obtained from the regression model. The reference line in the graph represents the ideal situation where the actual values correspond exactly to the predicted values. The concentration of points near this line visually confirms the high accuracy and reliability of the proposed model.

When monitoring the dependence of deep melt penetration along the outer walls of the sample on the amount of KCl in the melt, we can speak of a very strong indirect linear dependence (*r* = –0.983).

#### 3.3.2. Depth of Melt Infiltration into the Bottom and Side Walls of Samples

**Figure 8:**
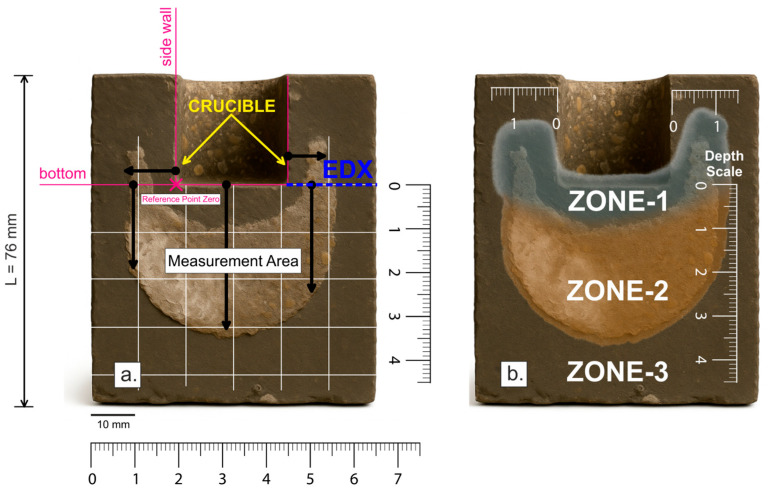
Schematic diagram of infiltration depth measurement with marked melt penetration zones. (**a**) marking of the measurement area with reference point zero; (**b**) infiltration zones.

Macroscopic analysis of the depth of melt infiltration was performed by measuring distances with a ruler with a maximum deviation of ±0.1 mm. The bottom or side wall of the crucible (crucible bottom: Ø: 25 mm, depth: 25 mm; see Figure 8a) was taken as the starting point of the coordinate system (reference point; *x* = 0). The distances measured along the positive direction of the x axis from this reference point quantify the depth of penetration of the corrosive agents (NaCl–KCl melt). Three characteristic zones were identified in each sample: (Zone-*1*) primary infiltration zone, characterized by corrosive processes; (Zone-*2*) secondary infiltration zone with detected chloride ion penetration; and (Zone-*3*) area without chloride penetration, representing the non-infiltrated zone.

Measurements taken from the bottom of the crucible showed significantly greater infiltration depth of the melt into the material. This phenomenon can be explained by the combined effect of gravity and melt accumulation at the bottom, leading to a more intense corrosion process. As a result, the depth of melt penetration was approximately 1.5 to 2 times higher compared to measurements taken from the side wall. Such increased infiltration depth in the bottom area can lead to faster weakening and premature failure of refractory materials.

Conversely, when measuring from the side walls, less penetration of the melting into the material was observed. Lower hydrostatic pressure acts in this area and there is also a lower concentration of melt, so the degradation process is slower. The depth of infiltration was therefore significantly less than in measurements taken from the bottom of the sample. Figure 8 provides a clear graphical representation and allows for a quantitative evaluation of the changes in alumina-silicate refractory materials (SiO_2_/Al_2_O_3_).

The measurements of the depth of melt infiltration into refractory samples (ASRM-*1* to ASRM-*5*) showed a significant dependence on the composition of chloride melts (see Table 6). For each sample, measurements were performed in two zones: Zone-*1* (primary zone), Zone-*2* (secondary zone) see Figure 8b. Zone-*3*, which represents an area without chloride penetration, was included in the chemical analysis. The exceptions were samples ASRM-*4* (20% KCl, 80% NaCl) and ASRM-*5* (100% NaCl), where data were obtained only for Zone-*2*. At high NaCl concentrations (≥80%), the melt penetrated almost the entire cross-section of the material (ASRM-*4*; ASRM-*5*), causing its structure to break down. As a result, Zone-*1* disappears, and its measurement is no longer possible. These results confirm once again that with increasing NaCl content, there is a sharp increase in the depth of melt penetration.

Analysis of the results confirms a systematic increase in infiltration depth with increasing NaCl content in the melt. When using pure KCl (100%), the lowest infiltration values were recorded: 4/5 mm in Zone-*1* and 13/26 mm in Zone-*2*, comparable to the extremely low infiltration (<0.1 mm) observed in low-porosity materials exposed to MgCl_2_–KCl–NaCl [6]. In contrast, with 100% NaCl, the highest infiltration values were measured—up to 18 mm on the side wall and 34 mm on the bottom in Zone-*2*. Intermediate compositions with mixed salt proportions (80/20%, 50/50%, and 20/80%) exhibited a progressive, continuous increase in infiltration depth. This trend clearly demonstrates the greater corrosive aggressiveness of NaCl compared to KCl; even a relatively small increase in NaCl content significantly raises infiltration depth. Several approaches can be used to reduce the risk of refractory material damage. First, the activity of chloride ions can be effectively limited by reducing the proportion of NaCl in the melt or by adjusting the oxidation-reduction conditions [30,31,32]. Second, the protective properties of the material can be enhanced by applying a low-porosity surface layer (“hot-face”) or by promoting the formation of a stable, magnesium-enriched surface layer that acts as a barrier against salt penetration [6,33]. These strategies provide an important basis for optimizing melt composition and selecting suitable protective measures, which can substantially extend the service life and improve the reliability of refractory materials under demanding corrosive conditions.

**Figure 9:**
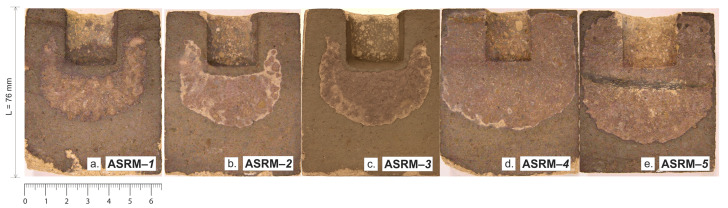
Macroscopic sections after corrosion testing in NaCl–KCl melt. (**a**) 100% KCl; (**b**) 80% KCl—20% NaCl; (**c**) 50% KCl—50% NaCl; (**d**) 20% KCl—80% NaCl; (**e**) 100% NaCl.

Figure 9 shows macroscopic sections of heat-resistant samples designated as ASRM-*1* to ASRM-*5* after corrosion testing in a NaCl–KCl melt. Each sample exhibits a characteristic semicircular area of material degradation caused by the action of molten salt. The varying intensity of corrosion observed on individual samples is the result of changes in the ratio of NaCl and KCl in the melt. Changes in the composition of the corrosive medium affect the chemical properties of the melt, such as the activity of individual ions, the melting point, and the ability to penetrate the pores of the material. A higher proportion of one of the components can lead to increased aggressiveness of the environment, which manifests itself in more intense surface degradation or greater infiltration depth of corrosion products. These differences provide a better understanding of how the specific ratio of NaCl and KCl affects the resistance of refractory materials under real operating conditions and can serve as a basis for optimizing the composition of the working environment or selecting the appropriate material for a given application.

#### 3.3.3. Microscopic Analysis

**Figure 10:**
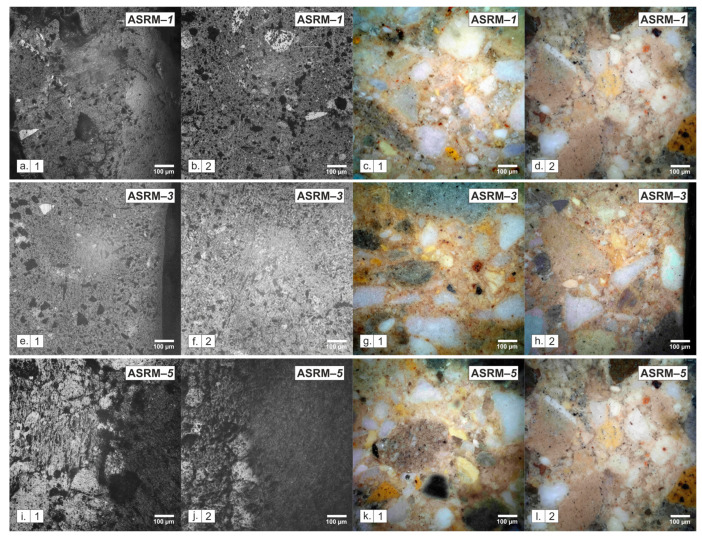
Microscopic sections after corrosion testing in NaCl–KCl melt at 25× magnification. The images show the infiltrated area (**1**) and the area without infiltration (**2**) of the melt: 100% KCl (**a**–**d**), 50% KCl—50% NaCl (**e**–**h**) and 100% NaCl (**i**–**l**), in reflected and polarized light.

Microscopic analysis served as a complementary method to macroscopic evaluation. Optical microscopy was performed in reflected and polarized light mode at a magnification of 25× (see Figure 10). This analysis provided additional information to support the interpretation of macroscopic corrosion manifestations but did not constitute the main source of conclusions in this study. Microscopic observation was performed in two areas of each sample, namely in the infiltrated area (1) and in the area without infiltration (2).

Figure 10 clearly shows the morphological differences between the infiltrated zone (1), which was directly exposed to the melt, and the original base material (2), which was not affected by the melt and is located at a greater depth of the cut. A comparison of the three compositions examined—pure KCl, a eutectic mixture of 50% KCl and 50% NaCl, and pure NaCl—confirms that both the intensity and nature of degradation are closely related to the chemical composition of the melt.

In reflected light mode, dark clusters of corrosive substances and fine pores appear in the infiltrated areas (Figure 10a.1,e.1,i.1). In contrast, in areas where no infiltration has occurred (Figure 10b.2,f.2,j.2), the material retains a more compact and uniform structure. Polarized light under a microscope allows the original parts of the material to be clearly distinguished from those that have been altered by chemical processes. The original mineral grains, such as silicates and corundum, appear in the image as light purple to blue or white-pink areas. These colors indicate that these parts have remained untouched and retained their original structure. Conversely, newly formed products resulting from the action of salt or other chemical changes appear as brownish yellow to gray fillings between the mineral grains. These areas show where the salt melt has penetrated and reacted with the material.

Pure KCl forms a thin, clearly defined corrosion layer with a thickness of about 150–250 µm. The eutectic mixture of NaCl–KCl, which melts at a lower temperature and is more fluid, penetrates deeper into the material. A transition zone 300–450 µm thick is formed. Pure NaCl causes the most damage to the material. It penetrates to a depth of 450–600 µm.

The observed differences in microstructure correspond to the macroscopic results of corrosion tests: the higher the NaCl content in the melt, the more pronounced the degradation of ASRM.

### 3.4. Chemical Analysis

This part of the research was focused on the chemical analysis of refractory materials (ASRM—SiO_2_/Al_2_O_3_). Using samples taken from different depths and areas of the material, we will monitor how the concentration of selected elements (Na, K, Cl, Si, Al) changes depending on the distance from the surface. The aim was to determine the depth of melt infiltration and assess how these processes affect the chemical stability of individual components of the refractory material.

**Figure 11:**
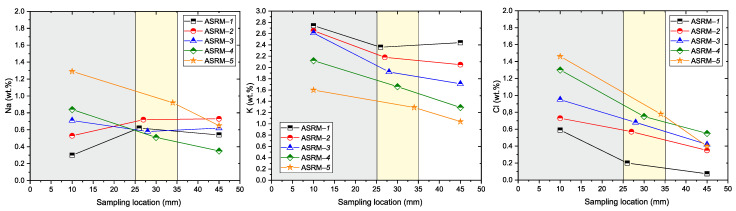
Concentrations of Na, K and Cl depending on the sampling location (gray—infiltrated area, yellow—border area, white—areas without infiltration).

**Figure 12:**
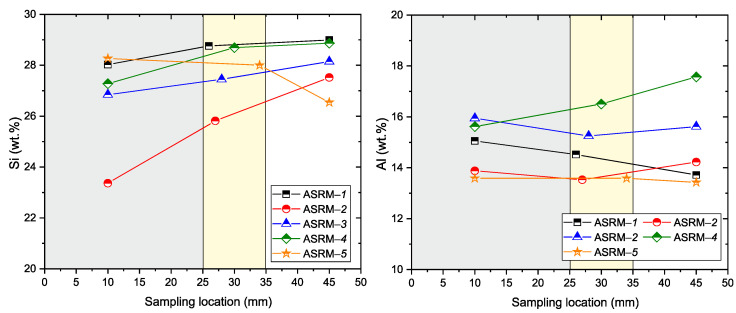
Concentrations of silicon (Si) and aluminum (Al) depending on the sampling location (gray—infiltrated area, yellow—border area, white—areas without infiltration).

To verify NaCl–KCl melt infiltration into the refractory material, chemical analyses were performed on samples collected from three areas (see Table 6): 10 mm, 25–35 mm, and 45 mm from the bottom of the crucible. The results are presented in Figure 11 and Figure 12.

Analysis of Na, K, and Cl elements (see Figure 11) reveals characteristic trends in the penetration of aggressive ions. Sodium (Na) and potassium (K) show maximum concentrations in the penetrated area (0–25 mm, gray), with their values decreasing significantly at the penetration boundary (25–35 mm, yellow) and becoming negligible in the unpenetrated zone (>35 mm, white). A similar trend is observed for chlorine (Cl), which has a maximum in the penetrated area and its concentration decreases rapidly towards the interior of the material. These results confirm the diffusion of sodium, potassium, and chloride ions from the surface layers of the material into its interior, with a infiltration depth of approximately 35 mm from the reference point *x* = 0 (bottom of the crucible: 25 mm). Figure 12 provides an overview of the degradation processes (Si, Al). Silicon (Si) shows the lowest values in the penetrated area, with its concentration increasing towards the non-penetrated zone. The decrease in silicon concentration in the penetrated area indicates possible silicon leaching, probably due to interaction with the aggressive environment. In contrast, aluminum (Al) maintains a relatively stable concentration in all zones of the material, confirming the higher chemical resistance of Al_2_O_3_ to corrosion compared to silicate phases.

Based on recent scientific publications, it can be concluded that the issue of penetration and corrosion of NaCl–KCl melt (or its mixtures with other chlorides) in refractory and metallic materials is being intensively investigated. Gage et al. [6] demonstrated that low-porosity refractory materials in the MgCl_2_/KCl/NaCl system significantly limit melt penetration and that the formation of stable secondary phases at the interface increases their chemical resistance. Similarly, authors [7] found that MgO and its mixtures exhibit high resistance to corrosion in NaCl, while Al_2_O_3_ and Cr_2_O_3_ are more susceptible to the diffusion of sodium and chloride ions into the surface layers. As demonstrated in [34], in the case of high-entropy alloys, selective dissolution of Fe and Cr occurs, accompanied by degradation of protective oxide layers and significant weight loss. The results of these studies confirm the importance of detailed analysis of chemical changes at different depth levels of the material, as well as the need to optimize the composition and microstructure of refractory materials to increase their service life in aggressive conditions.

### 3.5. Semi-Quantitative EDX Analysis

**Figure 13:**
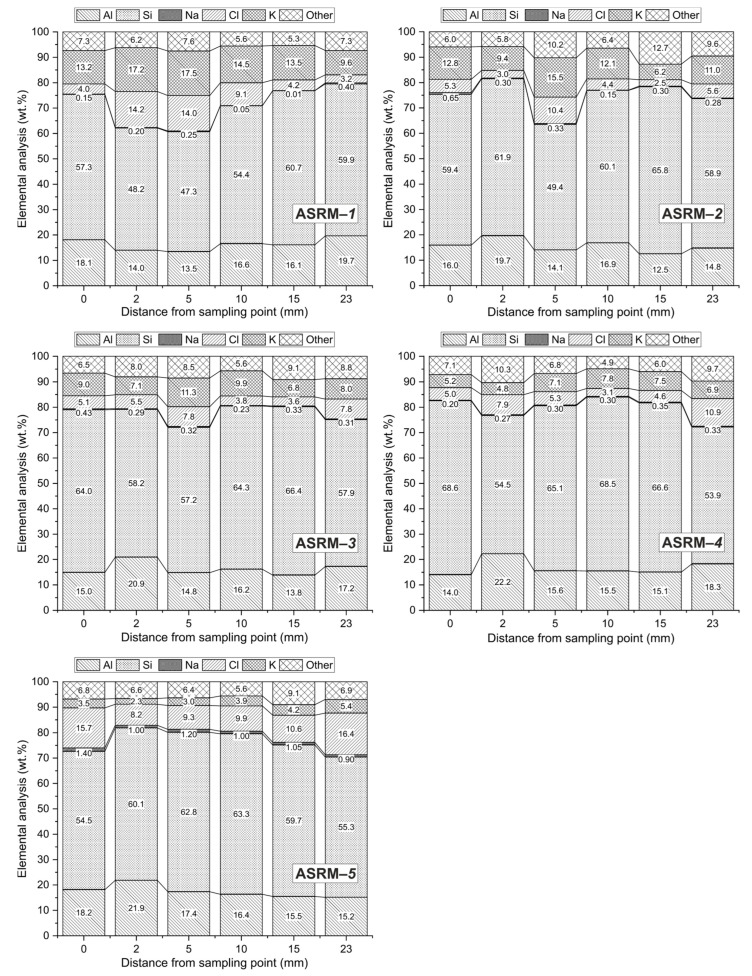
Changes in the content of elements in the profile of refractory samples (0–23 mm, EDX).

For a more detailed assessment of the corrosion process of alumina-silicate refractory materials under the influence of NaCl–KCl melts, a semi-quantitative local EDX analysis was performed on selected samples obtained by microscopic observation (Figure 8a; EDX; blue), a semi-quantitative local EDX analysis was performed using a JEOL 35CF electron microscope equipped with a LINK analyzer. The sampling location for analysis was selected according to the blue line shown in Figure 8a, which defines the transition zone between the undisturbed and corrosion-affected parts of the sample. This procedure ensured a detailed assessment of material changes within the transition zone and provided a comprehensive view of the nature of the corrosion processes occurring in the sample. The results of the semi-quantitative EDX analysis are shown graphically in Figure 13.

Local EDX microanalysis was performed on selected areas of the matrix with dimensions of at least 100 μm × 100 μm, representing the products of chloride melt corrosion. The correct selection of the analyzed area significantly influences the results of the semi-quantitative EDX analysis of these samples.

Silicon dioxide (Si) dominates in all analyzed samples, ranging from approximately 48 to 66% depending on the sample and measurement location. The aluminum (Al) content is stable in all cases, most often ranging from 14 to 18%. Sodium (Na), potassium (K) and chlorine (Cl) occur only in low concentrations, usually between 1 and 3%, with their values not varying significantly within the sample profile. The proportion of other elements is low and stable in all cases.

Although the concentrations of sodium, potassium, and chlorine are relatively low and stable in the overall sample profile, a more detailed EDX analysis revealed certain local differences in the distribution of chlorine ions. Based on the presented results, it can be concluded that a reaction layer is formed on samples ASRM-*4* and ASRM-*5*, with a thickness of approximately 2 mm. The highest concentration of chlorine ions in the impregnated area was found in samples ASRM-*1* and ASRM-*5*. A comparison of the chlorine content on the outer surface of the samples after the corrosion test shows that the highest values were measured in samples ASRM-*4* and ASRM-*5*. These findings correspond to the macroscopic observation of the infiltration depth depending on the composition of the melt. An increase in the NaCl content in the NaCl–KCl melt leads to an expansion of the infiltrated area of the masonry. The chlorine content in the products of the corrosion process also depends on its mass fraction in the respective compound—the mass ratio of Na:Cl in one mole of NaCl is 0.65, while in KCl the ratio is K:Cl = 1.103.

These results confirm that although the overall composition of refractory materials is chemically stable, local reaction layers may form, and chlorine may accumulate depending on the composition of the melt and the corrosion test conditions. This points to the need to optimize the composition of materials, especially in environments with higher NaCl content, where the risk of infiltration area expansion is more pronounced. Despite these local changes, however, none of the samples analyzed showed values that could be considered critical in terms of resistance to chemical degradation in NaCl–KCl melt environments. Based on these results, ASRMs are therefore suitable for use in aggressive environments where long-term stability is required.

## 4. Conclusions

Wear and corrosion of refractory materials in aluminum production and refining represent major challenges in materials engineering [35,36,37,38]. Because the high financial costs and technical complexity of testing under real operating conditions are prohibitive, scientific research focuses primarily on laboratory tests that enable detailed monitoring of degradation processes. At the same time, recent research points to the growing importance of environmental assessment of technologies and materials used in metallurgical production [39]. The most important degradation mechanism for refractories used in aluminum production and refining is chemical corrosion caused by inorganic salt melts, which serve as covering and refining mixtures [4,40]. The most commonly used salts include NaCl, KCl, CaCl_2_, and their mixtures. Their aggressiveness toward refractory materials depends on both their specific composition and operating conditions.

This study focuses on aluminosilicate refractories with a high SiO_2_/Al_2_O_3_ ratio, selected based on requirements from an industrial partner and their growing importance in aluminum metallurgy. Despite their industrial relevance, research on these materials remains limited. Our aim was to assess their resistance to chloride melts under conditions simulating real operating environments and identify the main factors affecting their service life.

Macroscopic analysis involved visual assessment of the extent of degradation and identification of regions penetrated by the melt, providing a basic overview of surface damage and the residual volume of material after exposure to aggressive salts. Microscopic analysis focused on monitoring microstructural changes resulting from melt exposure. Chemical analysis was used to quantify changes in the content of key elements—silicon (Si), aluminum (Al), sodium (Na), potassium (K), and chlorine (Cl)—at various depths within the material. This approach allowed precise determination of the extent and nature of chemical changes in refractory materials during corrosion testing. Local semi-quantitative EDX microanalysis enabled accurate mapping of corrosion product distribution within the microstructure, providing detailed information on element distribution in damaged regions.

Based on the experimental results and analyses, the following key conclusions can be drawn:

1. Linear regression modeling confirmed a very strong correlation between NaCl content and the degree of refractory material damage (*R*^2^ = 0.967, *r* = 0.983), allowing reliable prediction of corrosion behavior based on melt composition.
2. The lowest infiltration depth and melt loss were observed for pure KCl, while the highest values were found for pure NaCl. Even a small increase in NaCl content significantly increases corrosion aggressiveness and melt penetration.
3. Chemical analysis showed that silicon (Si) content decreases in the infiltrated zone, whereas aluminum (Al) remains stable. This indicates that Al_2_O_3_ exhibits greater corrosion resistance than silicate phases.
4. EDX analysis confirmed elevated sodium and chlorine concentrations in infiltrated regions, complementing AAS results and providing accurate spatial mapping of corrosion product distribution.
5. The greatest infiltration depth was observed at the bottom of the samples, attributable to the combined effects of gravity and melt accumulation. This region carries the highest risk of premature refractory failure.
6. Microscopic analysis confirmed that the intensity and nature of degradation are closely related to melt composition. Higher NaCl content leads to thicker, less adherent corrosion layers.
7. Reducing NaCl content in the melt, applying low-porosity surface layers, or creating protective barriers can significantly extend refractory service life in aggressive environments.
8. ASRMs are suitable for use with NaCl–KCl melts, but additional protective measures are recommended at high NaCl concentrations.
9. The results provide a quantitative basis for optimizing refractory composition and designing protective strategies to extend service life in aluminum metallurgy.

High-purity SiO_2_/Al_2_O_3_ refractory materials exhibit acceptable resistance to KCl-rich melts, but their service life decreases sharply with increasing NaCl content. A reliable linear infiltration model, together with chemical and microstructural characterization, provides a practical tool for optimizing the composition of both melts and linings and represents a starting point for targeted protection strategies in the metallurgical industry.

The methodological approach, which combined static crucible tests, multi-scale analytical techniques, and statistical modeling, enabled a comprehensive understanding of corrosion processes and provided a reliable means of predicting the service life of linings under real aluminum production conditions. Future research should focus on the influence of cyclic temperature loading, changing oxygen partial pressure (pO_2_) values, and testing functionally graded or nanocomposite refractory materials that could further extend the service life of linings in environments with high NaCl content.

## Figures and Tables

**Table 1:** Phase and chemical analysis of alumina-silicate refractory materials.

Declared/Measured Value	Analytical Method	Phase Analysis (wt.%)							
		SiO_2_	Al_2_O_3_	Fe_2_O_3_	TiO_2_	CaO	MgO	Na_2_O	K_2_O
Manufacturer ^1^	–	68	26	<2.5	–	–	–	–	–
Laboratory ^2^	XRD ^3^	65	26	2	1	1.5	1	0.29	1.13
**Declared/Measured Value**	**Analytical Method**	**Chemical Analysis (wt.%)**							
		**Si**	**Al**	**Fe**	**Ti**	**Ca**	**Mg**	**Na**	**K**
Manufacturer ^1^	–	31.77	13.76	<1.75	–	–	–	–	–
Laboratory ^2^	AAS ^4^	29.43	13.76	1.59	0.59	1.08	0.60	0.22	0.94

^1^ Manufacturer—data declared by the manufacturer; ^2^ Laboratory—data measured in the laboratory; ^3^ XRD—X-ray Diffraction; ^4^ AAS—Atomic Absorption Spectroscopy.

**Table 2:** Physical and mechanical properties of refractory materials.

Property	Unit	Average ± SD (*n* = 5) ^1^	Declared Value ^2^
Cold compressive strength	MPa	45 ± 2	60
Bulk density	kg/m^3^	2130 ± 7.7	2150
Apparent porosity	%	17 ± 1.3	14
Water absorption	%	8.1 ± 0.58	–
Maximum operating temperature	°C	–	1100

^1^—data measured in the laboratory; ^2^—data declared by the manufacturer.

**Table 3:** Comparison of the properties of NaCl and KCl.

Properties	Designation	Unit	NaCl	KCl
Density	*ρ*	g/cm^3^	2.165	1.984
Molar mass	*M*	g/mol	58.443	74.551
Molar volume	*V_m_*	m^3^/mol	2.699 × 10^−2^	3.757 × 10^−2^
Melting point	*t_top_*	°C	800	771
Boiling point	*t_var_*	°C	1413	1420
Surface tension	*σ*	mN/m	113.8	97.4

**Table 4:** Melt loss and corrosion parameters for ASRM samples.

Sample	KCl–NaCl (wt.%)	KCl (mol.%)	NaCl (mol.%)	Melt Loss (wt.%)	Temperature (°C)	Time (h)
ASRM-*1*	100–0	100.00	0.00	33.34	801	96
ASRM-*2*	80–20	83.60	16.40	34.18		
ASRM-*3*	50–50	56.00	44.00	36.15		
ASRM-*4*	20–80	24.20	75.80	38.72		
ASRM-*5*	0–100	0.00	100.00	40.00		

**Table 5:** Parameters of the regression model.

Parameter	Point Estimation	95% Confidence Interval	
		Lower Limit	Upper Limit
*β*_0_	51.237	49.756	52.719
*β*_1_	0.0698	0.046	0.094

**Table 6:** Overview of the results of NaCl–KCl melt infiltration in zones and measurement locations.

Sample	NaCl–KCl Melt	Zone of Measurement	Place of Measurement	Depth of Infiltration (mm)
ASRM-*1*	100% KCl	Zone-*1*	Side wall	4
			Bottom	5
		Zone-*2*	Side wall	13
			Bottom	26
ASRM-*2*	80% KCl 20% NaCl	Zone-*1*	Side wall	3
			Bottom	4
		Zone-*2*	Side wall	11
			Bottom	27
ASRM-*3*	50% KCl 50% NaCl	Zone-*1*	Side wall	2
			Bottom	1
		Zone-*2*	Side wall	15
			Bottom	28
ASRM-*4*	20% KCl 80% NaCl	Zone-*2*	Side wall	20
			Bottom	30
ASRM-*5*	100% NaCl	Zone-*2*	Side wall	18
			Bottom	34

## Data Availability

The original contributions presented in the study are included in the article, further inquiries can be directed to the corresponding author.
